# Adapting power calculations to include a superiority margin: what are the implications?

**DOI:** 10.11613/BM.2024.010101

**Published:** 2024-02-15

**Authors:** Samuel Bishara

**Affiliations:** Division of surgery, West Middlesex Hospital, Chelsea and Westminster NHS Trust, United Kingdom

**Keywords:** biostatistics, power calculation, super-superiority margin, clinical significance, hypothesis testing

## Abstract

This paper examines the application of super-superiority margins in study power calculations. Unlike traditional power calculations, which primarily aim to reject the null hypothesis by any margin, a super-superiority margin establishes a clinically significant threshold. Despite potential benefits, this approach, akin to a non-inferiority calculation but in an opposing direction, is rarely used. Implementing a super-superiority margin separates the notion of the likely difference between two groups (the effect size) from the minimum clinically significant difference, without which inconsistent positions could be held. However, these are often used interchangeably. In an audit of 30 recent randomized controlled trial power calculations, four studies utilized the minimal acceptable difference, and nine utilized the expected difference. In the other studies, this was unclarified. In the *post hoc* scenario, this approach can shed light on the value of undertaking further studies, which is not apparent from the standard power calculation. The acceptance and rejection of the alternate hypothesis for super-superiority, non-inferiority, equivalence, and standard superiority studies have been compared. When a fixed minimal acceptable difference is applied, a study result will be in one of seven logical positions with regards to the simultaneous application of these hypotheses. The trend for increased trial size and the mirror approach of non-inferiority studies implies that newer interventions may be becoming less effective. Powering for superiority could counter this and ensure that a pre-trial evaluation of clinical significance has taken place, which is necessary to confirm that interventions are beneficial.

## Graphical abstract

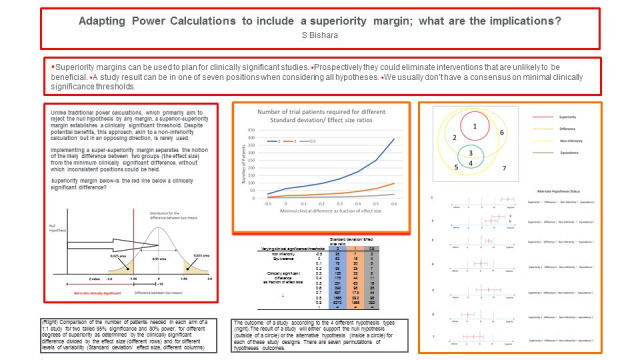

## Highlights

• Superiority margins can be used to plan for clinically significant studies

• Prospectively they could eliminate interventions that are unlikely to be beneficial

• A study result can be in one of seven positions when considering all hypotheses

• We usually don’t have a consensus on minimal clinically significant thresholds

## Introduction

Statistical power calculations for clinical trials are an integral part of planning, recruitment, and the financial costing of a study. Clinical studies do not always yield the expected results for a variety of reasons. In theory, if the statistical power (1-β) is set at 0.8 or 80%, this would be β times or 20% of the time (the type 2 error or false negative rate – failure to demonstrate a real difference in the study). The value of a study is that the answer is unknown beforehand. If it were, then there would be no merit in running the study. Power calculations are based on numbers that are not fully known and in essence will never be fully known, but for which there is some limited understanding, based on prior estimates. A further consideration for a study is whether the result is clinically significant, which statistically speaking is not bargained for in a standard power calculation. This paper explores the value of an alternative approach for power calculations, for superiority studies for continuous variables and its application in pre-study and *post hoc* scenarios.

Powering for clinical significance or a super-superiority margin is rarely undertaken. A search on Google Scholar for “super-superiority margin”, the margin for power for clinical significance, was carried out on the 24^th^ June 2023, yielding only 10 results, of which 3 are randomized controlled trials. By way of contrast, a search for “non-inferiority margin” yielded 12,900 results.

## Power for unpaired continuous variables

A pre-study power calculation aims to determine the number of people required in a study involving two groups that are being compared. For example, one might be the control group, which is receiving a placebo, no treatment or a conventional treatment and the other group, the test group, is receiving a new treatment. The aim of the study is to prove that a difference between the two groups is likely, and this is based on the observations that will be acquired in the planned study. A difference is proven by rejecting the null hypothesis or H_0_ - that there is no difference between the two groups, and accepting the alternative hypothesis H_1_ - there is a difference between the two groups. This can never be achieved with 100% confidence. Conventionally, 95% confidence is the most used threshold, so when the study is run, H_1_ is accepted if there is a ≤ 5% chance of H_0_ being true based on the study observations. This 5% threshold is the α (or type 1 error), the probability of observing a difference and declaring it to be real when there is no real difference between the two groups. It is conventional to use a two-tailed approach, treating differences between means in both directions with equivalency. A two-tailed approach splits the error into the upper and lower 2.5% of the distribution and raises the threshold for proving the H_1_ hypothesis slightly.

**Figure 1:**
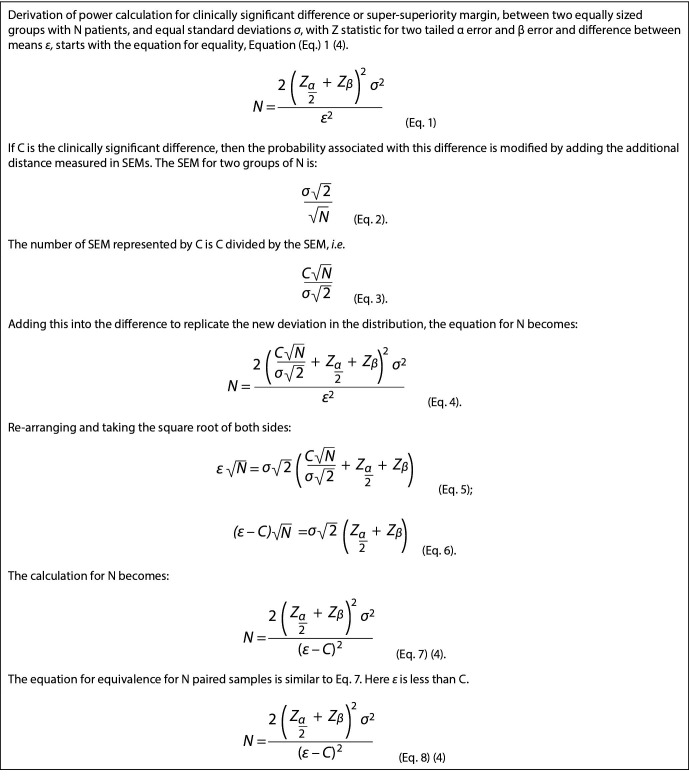
Derivation of power for equation for super-superiority margin for an unpaired t-test (*4*). SEM - standard error of the mean.

The calculation is based on the expected mean value of the continuous variable in each group, which is anticipated to be modified by the intervention that is being tested. The difference between these two means is the effect size. For example, if blood pressure (BP) is being treated (reduced) and the mean systolic blood pressure in the control is 150 mmHg and in the test group it is 140 mmHg, then the effect size is 150 mmHg - 140 mmHg = 10 mmHg. This effect size is usually regarded as the best available estimate of the likely difference between the two groups but is sometimes regarded as the minimal acceptable clinical difference or in some cases as a desired difference for the study (*1*-*3*). It is necessary to define the variability of blood pressure in the control and test populations. To simplify matters, it is sometimes assumed to be equal between the two groups. It is assumed in the following example where the variability of blood pressure, as measured by the standard deviation, is taken to be 20 mmHg. When designing a study, the ratio in which patients are distributed between the two groups is chosen. The most used ratio is an equal distribution between the two groups or 1:1, but other ratios are equally permissible. In terms of aiming to disprove the null hypothesis with the fewest overall number of patients, 1:1 is optimal, though there may be advantages to having a larger number of patients in an active group if this provides additional information for secondary outcomes, *e.g.* the tolerability of a drug. The α error and the β error need to be specified as described above. 1-β is the statistical power or the proportion of times that H_0_ would be rejected at the specified α level if the test was run many times. In the above worked example, assuming 1:1 recruitment with an α at 5% two-tailed, β at 20%, and equal population variance, using Equation 1 (Eq. 1, Figure 1): 2 x (1.96 + 0.84)^2^ x 20^2^ / 10^2^ = 63 patients would be needed in each group.

**Figure 2:**
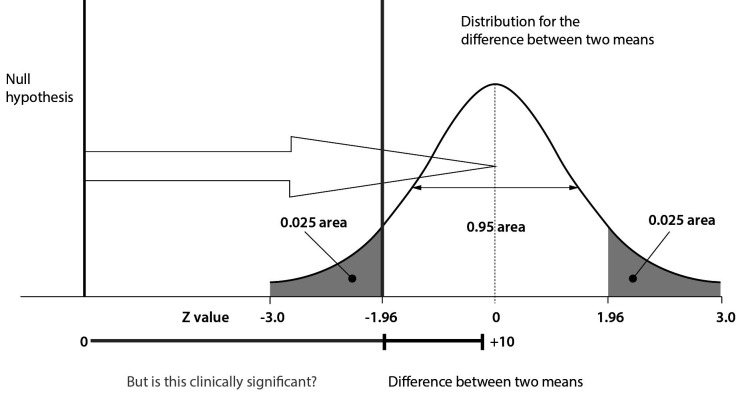
Graphical representation of super-superiority margin. This figure shows the typical results from a study comparing two normally distributed groups and the distribution for the difference between the means. In this example, a mean difference of 10 was achieved. The Z statistic shows the number of standard errors of the mean away from the mean with the 95% confidence interval. The null hypothesis has clearly been excluded (black vertical line) as this fall outside the confidence interval. However, every difference between the black vertical line and the grey vertical line has also been excluded, and we can ask if these values are clinically significant, as a value for the difference just greater than zero (the null hypothesis) may not be clinically significant. If we call the grey distance the minimally acceptable clinical distance C, then we can recalculate the t-test to determine the probability of obtaining this result if there was no difference between the two groups. So now, the z-value for this test equals the 1.96 for 95% CI (two-tailed) plus C. C needs to be converted into a z-value by dividing C by the SEM. For two groups with the same SD and sample size N, the SEM is SD x √2 / √N. The z-value for C is C √N / (SD x √2) and the z-value for this probability is 1.96 + C, *i.e.* 1.96 + C √N / (SD x √2). The equation simplifies as shown in Figure 1, Eq. 7, as N is on both sides of the equation. CI - confidence intervals. SD - standard deviation. SEM - standard error of the mean.

However, this approach to power calculations only caters to dispelling the null hypothesis, where any proven difference between the two groups is acceptable. A statistically significant difference between the two groups is not necessarily clinically significant. In many situations, statistically significant differences are also clinically significant, as there may not have been interest in conducting a study if the expected difference between the groups, based on prior data, is very small. The results of the study can be represented visually, as shown in Figure 2, which depicts the difference between the means and the probability distributions for the expected value of this difference with the 95% confidence interval. The null hypothesis has been rejected, as the confidence interval does not cross the zero-difference point. More than the null hypothesis has been excluded; everything outside of the 95% confidence interval has been excluded at the 5% level. This includes the null hypothesis and the distance between the null hypothesis, the 0 point, and the edge of the 95% confidence interval. This difference has been denoted by the letter C in the diagram, and we can query if C is clinically significant, as barely rejecting the null hypothesis would not demonstrate clinical significance with 95% confidence. Instead of designing a study to reject the null hypothesis, a study to reject non-significant superiority could be designed.

## How to design a clinically significant superiority study?

**Figure 3:**
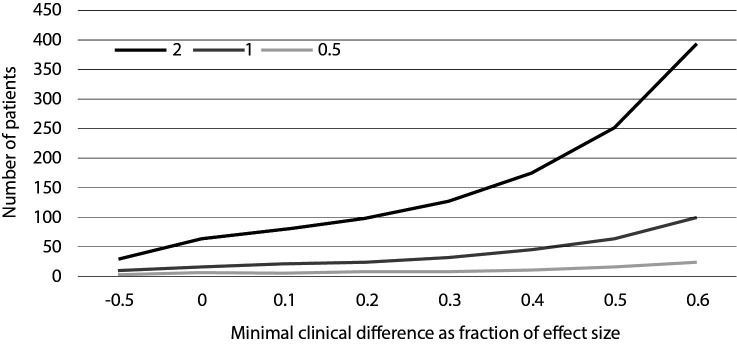
Number of trial patients required according to the size of minimal clinically significant difference as a fraction of the effect size. From Table 1 graphical representation of the number of trial patients required depending on the relationship between the effect size and the standard deviation (separate curves) and the minimal clinically significant difference as fraction of the effect size (x -axis).

A clinically significant superiority study could be one-tailed or two-tailed, but as a difference is specified in a particular direction, it may be reasonably conducted as a one-tailed argument. If using the 95% threshold as a two-tailed argument, the null hypothesis is associated with a z score of 1.96, which is to say that the observed difference is ≥ 1.96 standard deviations away from the mean (this is the effect size divided by the standard error of the mean (SEM = SD √2 / √N) where N is the number of people in each arm of the study (1:1)). C needs to be expressed in standard deviations by dividing it by the SEM. For example, if the clinical significance level C is equal to 1 SEM, this is added to 1.96 to yield a z value of 2.96 and the power calculation is reset for a z value of 2.96. From a z distribution table, this is associated with a probability of 0.3%, which is similar to the probability of a two-tailed test for the null hypothesis at the 0.3% level. Therefore, in this example, testing a superiority of 1 SEM at the 5% level is the same as testing the null hypothesis at the 0.3% level. The derivation of the equation is shown in Figure 1. Table 1 and the associated Figure 3 depict the number of patients needed in each arm of a study with 1:1 recruitment for testing a two-tailed hypothesis at the 95% level with 80% power. This is shown for different values of clinically significant value C expressed as a ratio of the effect size, and for different variabilities expressed as the ratio of the SD over the effect size.

**Table 1:** The effect of varying super-superiority margins on the number of trial patients required

		**Standard deviation/effect size ratio**		
Varying clinical significance thresholds		2	1	0.5
Non inferiority	- 0.5	28	7	2
Equivalence	0	63	16	4
Clinically significant difference as fraction of effect size	0.1	78	20	5
	0.2	98	25	7
	0.3	128	32	8
	0.4	175	44	11
	0.5	251	63	16
	0.6	392	98	25
	0.7	697	175	44
	0.8	1568	392	98
	0.9	6272	1568	392
	1	∞	∞	∞
Comparison of the number of patients needed in each arm of a 1:1 study for two tailed 95% significance and power at 80% for different degrees of superiority as determined by the ratio of the clinically significant difference as a fraction of the effect size (different rows) for different levels of variability (as determined by the ratio of the Standard deviation/ effect size, different columns). For small studies, the numbers are not accurate as a z statistic is used throughout rather than the t statistic.				

If the clinically significant value (C) = 0 this results in the standard power equation for equivalence; if a negative number is chosen for the difference, then this becomes a non-inferiority equation. It is impossible to prove a difference greater than or equal to the actual effect size as the number of people needed tends towards infinity, as the clinically significant difference approaches the actual difference. This is because, as the number of patients increases, SEM decreases, and the difference expressed as the number of SEM becomes ever larger.

The hypotheses are rewritten as: H_1_ - a greater than or equal to 95% chance that there is a greater than C difference between two groups; and, H_0_ - a greater than C difference has not been demonstrated with greater than or equal to 95% certainty.

## *Post hoc* power analysis

Power analysis is used to determine the design of a planned study but has sometimes been applied retrospectively to a study that has taken place. Often this is where there has been a negative result, where the null hypothesis was upheld, and sometimes at the request of journal editors. *Post hoc*, though a power value can be calculated, it does not provide any additional information beyond the P-value obtained in the study (*5*, *6*). If a study just achieves statistical significance at 95%, for example, when it is repeated many times, based on the parameters obtained from this study, it would be expected that these planned studies average around the values obtained in the first study, and it would achieve statistical significance 50% of the time which is to say that the statistical power is 50%. The calculated power is purely dependent on the P-value alone, and all other observed study parameters are redundant in this calculation. However, there is merit in looking into the study parameters of a negative study, as the acquired data will become a significant proportion of the available information on the clinical question. The question is not what is the power of that study but where do we go from here? Do we need another study? How many people need to be recruited, and how much will it cost?

Broadly speaking, there are three reasons why the expected result was not achieved despite sufficient people being recruited; either 1) the observed difference was less than expected, 2) the variance was greater than expected, or 3) a combination of the two. Is there a difference between scenarios 1 and 2? This is explored in the following example. A study examining a BP medication was expected to yield a mean difference in BP of 20 mmHg with a SD of 10 mmHg. What if the study yielded a difference of the means of 20 mmHg and the SD was 20 mmHg (scenario 1) *versus* a study with a difference of means of 10 mmHg with an SD of 10 mmHg (scenario 2). In both cases, if a power calculation for disproving the null hypothesis is carried out, 16 patients would be required in each group (Figure 1, Eq. 1). However, if instead of asking for the null hypothesis to be disproved, we are asking for a clinically significant difference, say of ≤ 5 mmHg, to be disproved. Scenario 1 would require fewer patients, and this is true for any clinically significant value > 0 (but less than the real difference). Although in both cases the ratio of the difference of means to the SEM is the same, as the actual clinical difference of 10 mmHg in scenario 1 is the same number of standard deviations away from the mean as 5 mmHg in scenario 2, a change of 10 mmHg is more clinically significant than a change of 5 mmHg. If a power calculation for a difference of 5 mmHg is carried out, in scenario 1, 28 people (Figure 1, Eq. 7; (2 x (1.96 + 0.84)^2^ x 20^2^ / (20-5)^2^) would be needed in each group and in scenario 2, 63 people ((2 x (1.96 + 0.84)^2^ x 10^2^ // (10-5)^2^) would be required in each group (power of 80%, α = 5% (two-tailed for both). Thus, a more clinically significant result is likely in scenario 1, but this is only apparent if a hypothesis for superiority rather than the null hypothesis is tested.

## Power calculation for equivalence

The power calculation for equivalence is closely related to a super-superiority margin power calculation. However, in trying to prove equivalence, the real difference must be less than the minimal acceptable clinical difference, unlike a superiority calculation where the real difference must be greater than the minimum acceptable clinical difference. If we take a laboratory test as an example, we may be comparing two blood glucose analyzers to determine if the bias or systematic error between them is less than an acceptable threshold. Using Figure 1, Eq.8, which is for a paired t-test as each sample could be split between the two analyzers. If the acceptable difference is 0.5 mmol/L, we could postulate that the actual difference was 0.2 mmol/L, and there is a common standard deviation of 0.5 mmol/L between the two analyzers. We could husk how many samples would we have to run to demonstrate that the bias was less 0.5 mmol/L at two tailed α at 5% and β at 20%. N = (1.96 + 1.28)^2^ x 0.5^2^ / (0.5-0.2)^2^ = 30 test samples. Proving this hypothesis for equivalence does not mean that the analyzer results are sufficiently accurate as the random error (standard deviation) could be greater than desired (*7*).

## The four different possibilities for trial design

A classic superiority design is by far the most common approach taken in clinical trials. In a sense, this is the purest study design as it requires no arbitration in setting a clinical difference, but some effect size has already been broached when undertaking a power calculation. It is also necessary to contextualize the effect size in terms of its value to patients. In powering a study, either the expected difference or the minimum clinical difference is utilized. Using an expected difference can lead to an avoidance of addressing the question as to whether this measured effect is likely to be of benefit to patients. The CONSORT guidelines recommend that a justification of the effect size is required in all power calculations, giving the example of the minimum acceptable effect size (*8*). For all the controversy regarding non-inferiority studies and the possibility of reducing the threshold for new therapeutic interventions, it does at least lead to an appreciation of the minimum effect size.

**Figure 4:**
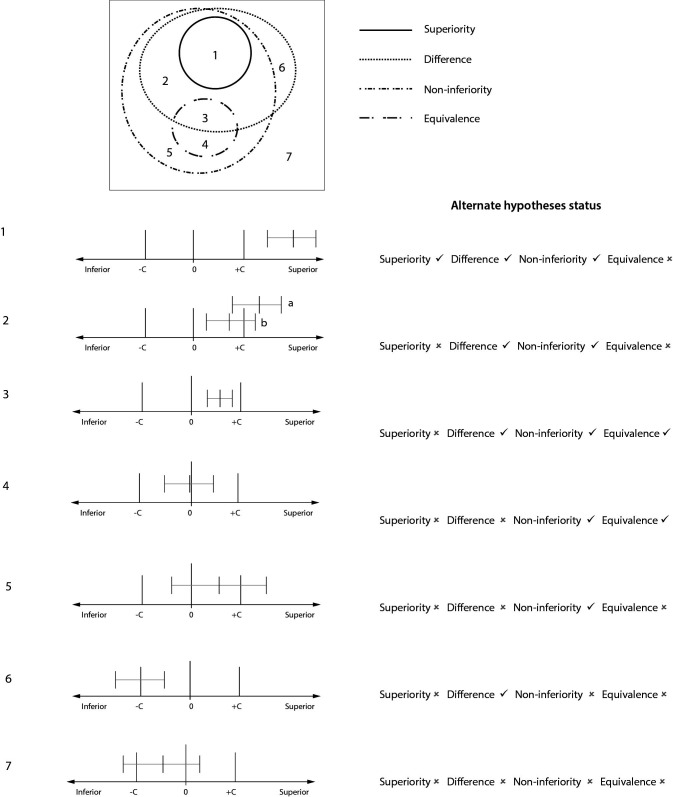
The outcome of a study according to the four different hypothesis types. The diagram shows the particular incidence of having the same minimal clinical significance threshold for a superiority, equivalence and non-inferiority design, then there are seven permutations of hypotheses outcomes when these hypotheses are applied simultaneously to a study outcome. The result of a study will support either the null hypothesis (outside of a circle) or the alternative hypothesis (inside a circle) for each of these study designs. This is further illustrated in the plots below where the mean and the 95% confidence interval (gray horizontal bar) for different study outcomes are shown against the different thresholds.

There are four possible trial designs: 1) the classical superiority study for difference; 2) non- inferiority; 3) equivalence; and 4) power for super-superiority or clinical difference. Thinking about how these hypotheses fit together can be confusing. These are compared in Figure 4. When considering a minimum effect size then all four of these hypotheses are available, and the figure explores where a study outcome lies when all four alternative hypotheses are applied simultaneously.

The result of a study will either support the null hypothesis or the alternative hypothesis for each of these study designs. The set inside of a hypothesis circle means that the alternate hypothesis for that design has been upheld, while lying outside the circle means that the null hypothesis has been upheld for that study design. If differing clinical significance thresholds are permitted, then the equivalence hypothesis is independent of the three other hypotheses, and 10 permutations of hypotheses outcomes are possible for a study. The diagram shows the particular incidence of having the same minimal clinical significance threshold for a superiority, equivalence, and non-inferiority design; then there are seven permutations of hypotheses outcomes.

In all cases, if superiority has been demonstrated, then a difference has already been demonstrated. If a difference has been demonstrated, then non-inferiority has been achieved unless the difference is in a larger inferior direction (region 6). Superiority and equivalence are mutually exclusive. The null hypothesis could be true with all the study designs (region 7). In outcome 2, where a difference has been shown (but not superiority) in the desired direction, if result a was obtained, then a clinically significant effect would be declared to be demonstrated. In the case of b, where the mean falls below the clinical superiority margin, a less than clinically significant difference was demonstrated but the difference between the two findings may be due to a high probability of chance and not demonstrated to a > 5% level in 1. In region 3, though a difference has been statistically demonstrated, a clinically significant difference has been excluded with > 95% certainty. In the previous example of a blood glucose analyzer the aim was for the study outcome to occupy region 3 or 4 in this diagram. The aim of powering a study for clinical difference is to make it more likely than not that the study will demonstrate an outcome compatible with region 1 in the diagram.

## Audit of recent randomized controlled studies

An audit of 30 recent randomized studies was carried out to determine the usage of power calculations (*9*-*38*). A PubMed search was performed on the 25^th^ June 2023, searching for BMJ (to bring up articles published in the BMJ Journal group) and adding the randomized controlled trials (RCT) filter, listing articles from newest to oldest. Articles were excluded if they were pilot studies, feasibility studies, study protocols, or not RCTs. Thirty articles were evaluated. These were published online between the 22^nd^ October 2022 and 23^rd^ June 2023 inclusive. The audit is summarized in Table 2. Twenty-nine of the studies were classic superiority studies and one was a non-inferiority study. Twenty-eight of 30 articles had published power calculation. Of the 28 power calculations, four were based on a minimal acceptable difference, nine used an expected difference and 15 did not give a clear justification for the difference used. Sixteen studies were non-significant, and 14 were significant for their primary outcome. Of the four studies that used the minimal acceptable difference in their power calculation, one was non-significant, one was significant but greater than the minimal significant margin but not at the 95% confidence interval equivalent to location 2a on the diagram. The other two studies were significant, but the mean difference was less than the minimally clinical difference; one was in location 2b, while the other is clinically significant margin was excluded, being outside the 95% confidence interval for the adjusted primary outcome *i.e.* in location 3. The authors of this study justified the result as being clinically significant as more than 50% of those who received the intervention had a greater than minimal clinically significant response. This may suggest that the responses did not fit very well with a normal distribution, though parametric statistics were used (*9*).

**Table 2:** Summary of findings from audit of 30 recent randomized controlled trials

**Type of Study** **(N = 30)**	**Published power calculation** **(N = 30)**	**Power calculation difference justification (N = 28)**	**Statistically significant** **(N = 30)**
Superiority (N = 29)	Yes (N = 28)	Minimum acceptable (N = 4)	Yes (N = 14)
Inferiority (N = 1)	No (N = 2)	Expected difference (N = 13)	No (N = 16)
/	/	Not stated (N = 15)	/

Though this audit is a small sample, it suggests that trials do not perform as well as their power calculations predict, as trials were powered to 80% or 90%. Indicating that 24 to 27 of the studies would have been expected to achieve statistical significance. Assuming 80% power throughout and that the superiority margin is an estimate of the actual treatment difference, then the probability of 14 out of 30 studies or less achieving statistical significance is 1.1 x 10^-5^. The low rate for the adoption of the alternative hypotheses does perhaps provide some reassurance against publication bias, where only “successful studies” are selectively published.

## Discussion

There is some ambiguity in the literature regarding the meaning of the clinical difference that is tested by a power calculation. Sometimes it is interpreted as the most likely difference between the two study groups, and other times, the minimally acceptable clinical difference between the two groups.

If the expected difference is used without reference to a minimally acceptable clinical difference, then we may lose perspective of whether this difference is beneficial to patients. When the latter definition is applied to a power calculation, this can yield an incongruous outcome, as a statistically significant difference between groups could be heralded as a positive outcome, even if it were less than the pre-determined, minimal clinically significant difference. This could occur if the test variable standard deviation was less than anticipated or more people were recruited into the study than planned. Likewise, if the minimum acceptable effect is exaggerated beyond the real effect, then the power calculation will yield a smaller study, which may not prove to be statistically significant. If the minimum acceptable clinical difference used is less than the real difference, or the apparent real difference, then a power calculation would lead to a larger sample size being required. This could be argued to be beneficial or produce a more costly trial than required, depending on the perspective, as the only stated aim was to dispel the null hypothesis. Assigning two separate variables to the likely difference and the minimally acceptable difference removes this ambiguity.

Nonetheless, there remains a degree of leeway in interpretation, particularly in the realm of continuous variables, which are largely surrogate markers for a qualitative or binary outcome. *E.g.*, BP control has no merit but for its reduction in the risk of cerebral vascular accidents or coronary events. Continuous variables may also be the most appropriate measure for chronic diseases where no cure is possible.

A possible reason for the ambiguity of usage of the power calculation is that the minimal clinically significant effect size may not have been previously addressed and perhaps isn’t really known (*39*). There is no standardized way for it to be determined (*40*). Strategies for determining the minimal clinically significant effect include looking at the degree of change that led to a change of practice in prior studies, the relationship between continuous variables and their binary consequences, and the associated economic cost-to-benefit ratio. These strategies require wider consultation with clinicians and patients (*41*). There may be no universal answer to what constitutes clinical significance, and it may differ between cultures and healthcare systems and change with time.

Outcome measures vary from those that are widely familiar to those which may be more niche, derived, or disease specific. These may not be as accessible to a wider readership. There is more reason in these instances for the minimal acceptable clinical difference to be defined. It is arguable that if the minimal acceptable clinical difference for an outcome is unknown, then aside from studies that measure survival, we won’t know if an intervention is beneficial to patients.

In binary outcomes, there are objective ways for quantifying the effect size, *i.e.* the number needed to treat (NNT) rather than the odds ratio, which is a relative risk reduction. The NNT is an independent means of comparing the effect size of different interventions, even in differing diseases, if an economic comparison is being made, for example. There is no exact equivalent for a continuous variable, but possible measures of absolute effect size are the percentage effect size, which is the effect size divided by the mean of the variable. In the above BP trial example, the percentage effect size would be 10/100, or 10%. An alternative to this is the effect size divided by the standard deviation (rather than the SEM, which is dependent on the number of patients in the trial). When assessing an intervention for a continuous variable among a subset who have a condition, if there is a clear demarcation between the disease subset and the ‘normal population’, then the degree of difference from the mean of the normal population has also been proposed as a basis of measuring clinical significance (*42*).

The calculation for a non-inferiority study is closely related to the calculations for superiority shown above and explores an alternative rationale for the adoption of a newer treatment. There may be some advantage to a new treatment other than the primary outcome, which has long been regarded as the bottom line, perhaps on cost grounds, convenience, or side effects. The ease of applicability of a new treatment is an important consideration that has gained increasing attention in recent years, addressing the likely implementation of a new intervention and the view that practicality and engagement should be early study considerations (*43*).

It also may have become unethical to employ a placebo as the comparator, and the threshold for patient benefit may have increased, and thereby the proof of superiority has become unattainable. However, the process of proving non-inferiority has lowered the threshold for acceptance of newer treatments when viewed solely on the performance of the primary outcome. Since the inception of randomized trials, there has likely been a gradual reduction in the clinical significance of newer treatments as it is becoming ever harder to find more effective therapies (*44*).

Another consequence of pursuing ever smaller clinical gains is that studies need to grow massively in size to demonstrate tiny benefits. This may make studies time-consuming, costly, and achievable only through commercial funding. While a large study may be viewed as highly informative, it may also be an indication that the intervention being tested is only of marginal benefit (*45*). When viewed over longer periods, the most effective interventions in medicine have often never been subjected to randomized trials, as their benefits are so obvious that any trial becomes unethical in some cases and difficult to recruit into in other cases (*46*). Examples of this are antibiotics and sterilization of operating fields. Even recent advances, such as the uptake of minimally invasive surgery, have not been subjected to randomized trials prior to change in practice and may never be (in their most current form) as the question may become irrelevant due to other technological advances (*47*). Progress through many tiny incremental gains may not be an effective use of resources and may be unnecessary when viewed as part of the overall development of medicine.

An alternative method of comparing two different hypotheses is the Bayes factor (*48*). The Bayes factor is the ratio of the likelihood of two competing hypotheses based on the observable data. To convert this into a probability, the Bayes factor is multiplied by the prior odds of one of the hypotheses being true based on prior observations or understanding. Typically, a ratio of > 3 or < 1/3 is deemed to be a significant effect. For example, if prior belief in the null hypothesis is 50% and the experiment yields a Bayes factor of 5, in favor of the alternative hypothesis, the prior odds are 1:1 and the posterior odds are 1 x 0.2:1. The probability is 0.2 / 1.2 = 0.17. The post-study null hypothesis probability in this example is 17%.

The Bayes factor is the ratio of the probability density for two different points on a distribution. A value of 6.7 is roughly equal to the ratio between the height of the midpoint of the normal distribution divided by the height when z = 1.96, which corresponds to a two-tailed 95% confidence interval. The Bayes factor contrasts with how probability is typically derived from a distribution. Conventionally, we calculate the area under the curve associated with a point and all points more extreme to it, be it ipsilateral (1-tailed) or bilateral (2-tailed), rather than a probability density at a particular point. The probability density is the limit of change in probability, as the change in z-value becomes negligible at a particular z-value.

A difficulty in exchanging a Bayes factor for a post-study probability, is how the pre-study probability of the null hypothesis is assigned with little or no prior data. The null hypothesis is the default hypothesis, and if an experiment shows no difference, when considering the area under the curve more extreme than the null hypothesis, then a probability of 1 is determined, as it encompasses the entire area under the curve if two-tailed. The alternative is to regard the null hypothesis exactly as an equality. The problem is that this is a very specific hypothesis, and at the limit of measurement, for two observations to be identical is impossible. For example, we might hypothesize that a child will be born at exactly 2 pm tomorrow. If we take the limit of measurement of time, which is currently possible (10^-20^ seconds) then we are highly unlikely to find a birth that takes place at that time, as precisely as we can measure it (*49*). Hence the rationale for the probability of continuous variables being determined as inequalities, *i.e.* the probability of observing a difference greater or equal to that observed, and proposals that the null hypothesis can be defined as an inequality, *i.e.* no difference or worse, rather than just no difference at all (*50*).

The study P-value is the probability that the study results would be obtained if the null hypothesis was true. It is inferred that this is also the probability of the null hypothesis, given the observed study results. As we know from an analogous binary situation, the sensitivity of a test (S), *i.e.* the probability of getting a positive test result given the presence of disease, is not interchangeable with the positive predictive value (PPV), the probability of someone with a positive test result having the disease (which is dependent on the incidence of the disease), *i.e.* the prior probability. The relationship between PPV and S is governed by Bayes theorem; PPV x (probability of a positive test) = S x (probability of disease = incidence) (*51*). However, because of the symmetry of the normal distribution, provided the assumption of normality holds true for the test variable, the two arguments are interchangeable. If a clinical effect of 4 units is found, then the null hypothesis is 4/SEM Z-statistics away from the center of the study distribution, and likewise, an effect of 4 units is 4/SEM away from the center of the null hypothesis distribution in the opposite direction. The test variable may be shifted across the x-axis of the distribution by changing the frame of reference (or scaled up or down, *e.g.* by changing the units of measurement), but the Z number and the associated area under the curve that they represent are unaltered. In this regard, there is nothing particular about the null hypothesis; it is just a hypothesis that occupies an area under the curve like any other hypothesis. Pre-study, the probability of the null hypothesis being falsely upheld is assigned the value β, which differs from α. The β value arises from considering the observed P-value as a binary variable to serve as a decision-making tool. The β-value is the probability that the observed P-value will be below the predefined α threshold, but post-study the probability of the null hypothesis is the observed P-value. Here, the null hypothesis is rejected, and an alternative hypothesis centered on the observed effect size is adopted.

In conclusion, a power for superiority calculation raises the threshold for carrying out a clinical study. This could increase the size of cost of the study but could also serve as a filter to eliminate studies that are unlikely to achieve results that are clinically beneficial. Reference to a minimal clinically significant difference is desirable in determining the power of all clinical studies, without which it is unknown if outcome differences are clinically beneficial. However further research into defining clinically significant differences is required, so there is greater standardization and clarity. *Post-hoc* analysis of power for superiority can help to determine the likelihood of obtaining clinical significance, which may be lost in a conventional power calculation, which yields no new information in the *post-hoc* setting.

## Data Availability

No data was generated during this study, so data sharing statement is not applicable to this article.
